# A potential protective element of myocardial bridge against severe obstructive atherosclerosis in the whole coronary system

**DOI:** 10.1186/s12872-018-0847-8

**Published:** 2018-05-29

**Authors:** Lisheng Jiang, Min Zhang, Hong Zhang, Lan Shen, Qin Shao, Linghong Shen, Ben He

**Affiliations:** 1 Department of Cardiology, Shanghai Jiao Tong University Affiliated Chest Hospital, Shanghai, China; 2 Department of Clinical Medicine, Shanghai Medical School, Fudan University, Shanghai, China; 3 Department of Cardiology, Renji Hospital, School of Medicine, Shanghai Jiaotong University, Shanghai, China; 4 Institution of Biostatistics, School of Life Science, Fudan University, Shanghai, China

## Abstract

**Background:**

Myocardial bridge (MB) is generally described as a congenital benign variation. Previous studies have suggested that MB prevents atherosclerotic plaques from accumulating within the bridge segment but promotes coronary stenosis in the proximal segment adjacent to MB. However, it is still not clear whether MB has positive or negative effects on severe obstructive atherosclerosis in the whole coronary artery system.

**Methods:**

In this study, 6774 patients with symptoms of angina who were clinically diagnosed coronary artery disease (CAD) or suspected CAD underwent coronary angiography (CAG) in our center. The presence of MB was diagnosed, and a retrospective analysis was performed between MB and severe obstructive CAD requiring percutaneous coronary intervention (PCI) or coronary artery bypass grafting (CABG) in the whole coronary system.

**Results:**

Among 6774 patients, 3583 (52.89%) were diagnosed with severe obstructive CAD (SOCAD) requiring a treatment of PCI or CABG and enrolled into the SOCAD group; and 3191 (47.11%) without SOCAD into the non-SOCAD group. Non-SOCAD and SOCAD groups had 512(16.05%) and 66(1.84%) patients with MB, respectively (*P* <  0.0001). The rate of SOCAD requiring PCI or CABG in patients with MB was much lower than that in patients without MB (11.42% vs. 56.76%, *P* <  0.0001). After adjusting for sex, age, diabetes mellitus, hypertension, and other risk factors, MB still had some positive role in preventing severe obstructive CAD (log-OR = − 2.134, *p*-value < 0.0001) through logistic regression.

**Conclusions:**

Our results provided a clue that MB might act as a potential protective element against severe obstructive atherosclerosis in the whole coronary artery system.

## Background

Myocardial bridge (MB) is referred to muscle overlying intramyocardial segment of an epicardial coronary artery, usually in the middle segment of the left anterior descending coronary artery (LAD) [1, 2]. Some studies reported anatomical properties of MB on atherosclerosis evolution in LAD. Location, length, and thickness are closely interrelated, and longer or thicker MBs are located significantly proximally in LAD [3]. Its characteristic compression of the tunneled coronary segment is clinically silent in many cases but is of interesting to clinical researchers due to its association with myocardial ischemia [4, 5].

The golden standard of MB diagnosis in angiography is defined as systolic milking effect produced by systolic compression by the intramyocardial segment [6]. MB is the most common congenital coronary variation, and the prevalence of MB varies from less than 5% [1, 6] under angiography, to 23% with intravascular ultrasound (IVUS) [6], to 55.6% under autopsy [7] due to the reason that short and thin bridges causing little systolic compression are easy to be ignored [8].

The presence of MB can be associated with various complications such as angina, acute myocardial infarction, arrhythmias, and even sudden death [4, 9–18]. MB can also be considered a benign variation of coronary arteries [19], or a double-edged sword [5]. The cause of angina is generally thought to be a distinct reduction of coronary artery flow due to muscular compression during systole [5, 20, 21]. Previous studies have suggested that in the intramyocardial segments, the vessel is protected from obstructive atherosclerosis, however, it is not clear whether MB has positive or negative effects on obstructive atherosclerosis in the whole coronary artery system. In the present study, we aimed at exploring a clinical relationship between MB and severe obstructive atherosclerosis requiring treatment with percutaneous coronary intervention (PCI) or coronary artery bypass grafting (CABG) in the whole coronary artery system.

## Methods

### Study oversight

This study is a retrospective observation based on hospital records from Renji Hospital, School of Medicine, Shanghai Jiaotong University, China. The authors assume responsibility for the accuracy and completeness of the data and data analyses.

### Data collection

From December 2012 to February 2015, 6774 patients with symptoms of angina who were clinically diagnosed with coronary artery disease (CAD) or suspected CAD underwent 6848 coronary angiographies in Renji Hospital. We conducted a retrospective study on MB by retrieving these patients’ hospital records, including sex, age, coronary risk factors, diagnoses of coronary angiography and invasive treatments. All clinical diagnoses follow the standard of ICD-10.

**Fig. 1:**
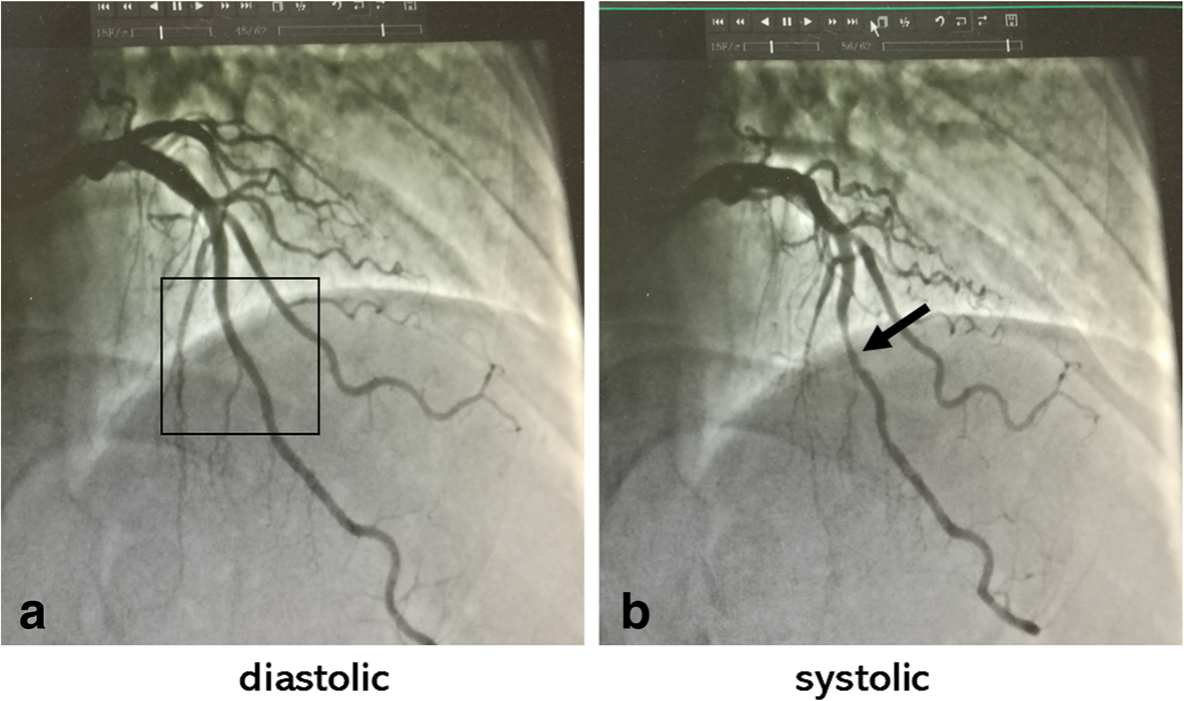
The typical characteristics of MB under angiography. The box in diagram **a** represents the segment of myocardial bridge free of compressing in left anterior descending artery during diastole; the arrow in diagram **b** represents the compressing segment of myocardial bridge in the same artery during systole

The presence of MB was recognized by the angiographic finding of transient reduction in the lumen of one epicardial coronary artery during systole as shown in Fig. 1. The severe obstructive coronary artery disease (SOCAD) requiring invasive treatment with PCI or CABG was defined as the presence of stenosis over 75% or occlusion in at least one major coronary artery, or stenosis less than 75% but over 50%, which was evaluated with an indication of PCI or CABG by coronary interventional cardiologist or cardiac surgeon. According to angiography results, patients with SOCAD underwent treatment with PCI or CABG and were enrolled into the SOCAD group; while patients without severe obstructive coronary artery lesion were enrolled into the non-SOCAD group.

The traditional risk factors including advanced age, hypertension, diabetes mellitus (DM) and impaired glucose tolerance (IGT), hyperlipidemia, chronic kidney disease (CKD), ischemic cerebrovascular disease (ICVD), etc. were documented to be linked with atherosclerosis. In the present study, both the incidence of MB and the risk factors as above were therefore recorded and analyzed.

### Statistical analyses

Mean values with standard deviations and counts with percentages were used to describe baseline characteristics and the incidence of MB. Differences were calculated separately in different subgroups according to presence or absence of SOCAD or MB, and sex. The differences were evaluated using one-way analysis of variance for continuous variables and Fisher’s exact test for categorical variables. The association between SOCAD and MB was further evaluated in the context of logistic regression model with or without interaction terms by adjusting for some baseline risk factors and the widely used stepwise variable selection strategy based on Akaike’s information criterion [22] was used to select those factors potentially associated with SOCAD.

All *P* values were two-sided, and a *P* value of < 0.05 was considered with statistical significances. The R program, version 3.4.0, was used to perform statistical analyses.

## Results

### Findings of myocardial bridge

As listed in Table 1, out of the 6774 patients underwent angiography, 578 (319 male and 259 female)were diagnosed with MB including 571 located in the left anterior descending artery (LAD), 4 in left circumflex (LCX) and 3 in right coronary artery (RCA).

**Table 1:** Clinical characteristics in patients with myocardial bridge (MB)

Characteristics	Values
Incidence of MB, n/total (%)	578/6774 (8.53%)
Location of MB: LAD^a^, n (%)	571(98.79%)
LCX^b^, (%)	4 (0.69%)
RCA^c^, (%)	3 (0.52%)
Age (years, mean ± SD)	61.10 ± 9.93
Sex	
Male, n (%)	319 (55.19%)
Female, n (%)	259 (44.81%)
Hypertension, n (%)	289 (50.00%)
DM^d^, n (%)	59 (10.21%)
IGT^e^, n (%)	22 (3.81%)
DM/IGT^f^, n (%)	81 (14.01%)
Hyperlipidemia, n (%)	42 (7.27%)
Ischemic cerebrovascular disease, n (%)	21 (3.63%)
Chronic kidney disease, n (%)	7 (1.21%)
SOCAD^g^, n (%)	66 (11.42%)

^a^LAD, left anterior descending artery

^b^LCX, left circumflex

^c^RCA, right coronary artery

^d^DM, diabetes mellitus

^e^IGT, impaired glucose tolerance

^f^DM/IGT, diabetes mellitus/impaired glucose tolerance

^g^SOCAD, severe obstructive coronary artery disease requiring treatment with percutaneous coronary intervention or coronary artery bypass grafting

### Incidence of MB and risk factors between patients with or without SOCAD

There were significant differences when comparing the incidence of MB between patients with or without SOCAD. As listed in Table 2, the incidence of MB in the SOCAD group was much lower than the non-SOCAD group (proportions: 1.84% vs. 16.05%, respectively; *P* <  0.0001). Besides, in the SOCAD group, there were older age (mean [±SD], 65.08 ± 10.55 years vs. 63.34 ± 10.33 years; *P* <  0.0001), higher proportion of male (74.83% vs. 52.74%, *P* <  0.0001), and higher rates of risk factors including, hypertension, diabetes and/or impaired glucose tolerance, chronic kidney disease and ischemic cerebrovascular disease. However, the rate of hyperlipidemia in the SOCAD group was lower than that in the non-SOCAD group, which might be linked with the reason that patients in the SOCAD group were given an intensive lipid-lowering therapy even before admission (some of them had a long history of coronary heart disease).

**Table 2:** Comparisons on incidence of myocardial bridge and risk factors in patients with or without SOCAD^d^

Event	Non-SOCAD	SOCAD	*P* value
	*N* = 3191 (47.11%)	*N* = 3583 (52.89%)	
Age (years, mean ± SD)	63.34 ± 10.33	65.08 ± 10.55	< 0.0001
Sex			
Male, n (%)	1683(52.74%)	2681(74.83%)	< 0.0001
Female, n (%)	1508(47.26%)	902(25.17%)	
Myocardial bridge, n (%)	512(16.05%)	66(1.84%)	< 0.0001
Hypertension, n (%)	1725(54.06%)	2377(66.34%)	< 0.0001
DM^a^, n (%)	527(16.52%)	1078(30.09%)	< 0.0001
IGT^b^, n (%)	124(3.89%)	197(5.50%)	0.0078
DM/IGT^c^, n (%)	651(20.40%)	1275(35.58%)	< 0.0001
Hyperlipidemia, n (%)	272(8.52%)	233(6.50%)	0.0016
Ischemic cerebrovascular disease, n (%)	178(5.58%)	259(7.23%)	0.0064
Chronic kidney disease, n (%)	83(2.60)	158(4.41%)	< 0.0001

^a^DM, diabetes mellitus

^b^IGT, impaired glucose tolerance

^c^DM/IGT, diabetes mellitus/impaired glucose tolerance

^d^SOCAD, severe obstructive coronary artery disease requiring treatment with percutaneous coronary intervention or coronary artery bypass grafting

### Incidence of SOCAD and risk factors between patients with or without myocardial bridge

As shown in Table 3, in comparison with the non-MB group, patients in the MB group had much lower rate of SOCAD requiring PCI/CABG (11.42% vs. 56.76%, *P* <  0.0001), higher rate of female (44.81% vs. 34.72%, *P* <  0.0001), younger age (mean [±SD], 61.10 ± 9.93 vs. 64.56 ± 10.49, *P* <  0.0001), and lower rates of risk factors including hypertension (50.00% vs.61.54%, *P* <  0.0001), impaired glucose metabolism including DM and IGT (14.01% vs. 29.78%, *P* <  0.0001), ischemic cerebrovascular diseases (3.63% vs. 6.71%, *P* = 0.0026), and chronic kidney disease (1.21% vs.3.78%, *P* = 0.0006), but not for hyperlipidemia (*P* = 0.934).

**Table 3:** Comparisons on incidence of SOCAD and risk factors in patients with or without myocardial bridge

Event	Without MB	With MB	*P* value
	*N* = 6196 (91.47%)	*N* = 578 (8.52%)	
Age (years, mean ± SD)	64.56 ± 10.49	61.10 ± 9.93	< 0.0001
Sex			
Male, n (%)	4045(65.28%)	319(55.19%)	< 0.0001
Female, n (%)	2151(34.72%)	259(44.81%)	
Hypertension, n (%)	3813(61.54%)	289(50.00%)	< 0.0001
DM^a^, n (%)	1546(24.95%)	59(10.21%)	< 0.0001
IGT^b^, n (%)	299(4.83%)	22(3.81%)	0.5441
DM/IGT^c^, n (%)	1845(29.78%)	81(14.01%)	< 0.0001
Hyperlipidemia, n (%)	463(7.47%)	42(7.27%)	0.934
Ischemic cerebrovascular disease, n (%)	416(6.71%)	21(3.63%)	0.0026
Chronic kidney disease, n (%)	234(3.78%)	7(1.21%)	0.0006
SOCAD^d^, n (%)	3517(56.76%)	66(11.42%)	< 0.0001

^a^DM, diabetes mellitus

^b^IGT, impaired glucose tolerance

^c^DM/IGT, diabetes mellitus/impaired glucose tolerance

^d^SOCAD, severe obstructive coronary artery disease requiring treatment with percutaneous coronary intervention or coronary artery bypass grafting

### Differences on incidence of MB and clinical characteristics between male and female

Compared with the male, the female patients had a higher proportion of MB (10.75% vs. 7.31%, *P* <  0.0001), much older age (66.09 ± 10.09 vs. 63.25 ± 10.56 years old, *P* <  0.0001), higher rate of hyperlipidemia (9.46% vs. 6.35%, *P* <  0.0001), but much lower SOCAD requiring PCI or CABG (37.43%% vs. 61.43%, *P* <  0.0001) (Table 4).

**Table 4:** Comparisons on incidence of myocardial bridge and clinical characteristics between male and female

Event	Male	Female	*P* value
	*N* = 4364 (64.42%)	*N* = 2410 (35.58%)	
Age (years, mean ± SD)	63.25 ± 10.56	66.09 ± 10.09	< 0.0001
Myocardial bridge, n (%)	319(7.31%)	259(10.75%)	< 0.0001
Hypertension, n (%)	2617(59.97%)	1485(61.62%)	0.1855
DM^a^, n (%)	1010(23.14%)	595(24.69%)	0.1274
IGT^b^, n (%)	221(5.06%)	100(4.15%)	0.2371
DM/IGT^c^, n (%)	1231(28.21%)	695(28.84%)	0.8594
Hyperlipidemia, n (%)	277(6.35%)	228(9.46%)	< 0.0001
Ischemic cerebrovascular disease, n (%)	258(5.91%)	179(7.43%)	0.0174
Chronic kidney disease, n (%)	178(4.08%)	63(2.61%)	0.0016
SOCAD^d^, n (%)	2681(61.43%)	902(37.43%)	< 0.0001

^a^DM, diabetes mellitus

^b^IGT, impaired glucose tolerance

^c^DM/IGT, diabetes mellitus/impaired glucose tolerance

^d^SOCAD, severe obstructive coronary artery disease requiring treatment with percutaneous coronary intervention or coronary artery bypass grafting

### Logistic regression

Association intensities (log-ORs) between risk factors and severe obstructive atherosclerosis requiring PCI or CABG were reported in Table 4. There was a strong negative linear relationship between MB and severe obstructive atherosclerosis (log-OR = − 2.134, *P* <  0.0001), and other significant risk factors (including interaction terms) included old age (*P* = 0.0025), female sex (*P* <  0.0001), hypertension (*P* <  0.0001), impaired glucose metabolism (*P* <  0.0001), hyperlipidemia (*P* = 0.0436), interaction term between age and sex (*P* <  0.0001), interaction term between age and impaired glucose metabolism (*P* = 0.0003), and interaction term between sex and hypertension (*P* = 0.0122).

A negative log-OR means a protective effect against severe obstructive atherosclerosis, and vice versa. Log-ORs of age, hypertension, impaired glucose metabolism, interaction term between age and sex, and interaction term between sex and hypertension were positive, while log-ORs of MB, female sex, hyperlipidemia, and interaction between age and glucose metabolism were negative. The log-OR of myocardial bridge was − 2.134, suggesting a potential protective element of MB against severe obstructive atherosclerosis requiring PCI or CABG (Table 5).

**Table 5:** Analysis of logistic regression (with interaction terms)

	log-OR^b^	Std. error	z value	*P* value
(Intercept)	−0.383	0.217	−1.762	0.0780
Age	0.010	0.003	3.019	0.0025
MB	−2.134	0.137	−15.545	< 0.0001
Female sex	−3.139	0.379	−8.285	< 0.0001
Hypertension	0.341	0.073	4.689	< 0.0001
Impaired glucose metabolism^a^	1.160	0.215	5.389	< 0.0001
Hyperlipidemia	−0.205	0.102	−2.018	0.0436
Ischemic cerebrovascular disease (ICVD)	−0.025	0.136	−0.183	0.8545
Chronic kidney disease (CKD)	0.254	0.146	1.735	0.0828
Age × Sex (female vs. male)	0.028	0.006	4.852	< 0.0001
Age × Impaired glucose metabolism^a^	−0.012	0.003	−3.628	0.0003
MB × ICVD	−12.698	179.070	−0.071	0.9435
Sex × Hypertension	0.301	0.120	2.507	0.0122
Sex ×Impaired glucose metabolism^a^	0.118	0.067	1.770	0.0767
Hypertension × Impaired glucose metabolism^a^	−0.134	0.071	−1.881	0.0600
Impaired glucose metabolism × ICVD	0.175	0.124	1.416	0.1568

^a^Impaired glucose metabolism including diabetes mellitus and impaired glucose tolerance

^b^log-OR: log-odds ratio. A negative log-OR means a protective effect against severe obstructive coronary artery disease (SOCAD) requiring treatment with percutaneous coronary intervention or coronary artery bypass grafting on the premise that the presence of SOCAD was coded as 1 and non-SOCAD was coded as 0

## Discussion

Currently, many studies consider MB as a contributing factor in myocardial ischemia, angina, myocardial infarction and arrhythmia [4, 9–17]. However, less atherosclerotic lesions are found in bridge segments in contrast to non-bridged coronary arteries [18, 23–27]. Limited proof indicates that compression by contracting myocardial muscles may provide some potential anti-atherosclerotic mechanisms linked with the release of anticoagulant and growth factors [18]. However, the overall protective or detrimental role of MB in the whole coronary system and knowledge on the mechanisms are still desired.

According to previous studies, formation of atherosclerotic plaque can frequently be found at segment proximal to the bridge, while the intramural segment is typically absent [18, 23], but not in all cases [26]. As supported by a morphological observation of cholesterol-fed rabbits, foam cells and modified smooth muscle cells have the same distribution on a cellar level with atheromatous plaques at proximal segments but not at intramural segments [25]. Also, endothelial cells proximal to MB were arranged in a pavement-like, polygonal and flat appearance because of a high sheer stress [27]. These pathologic changes in proximal segment may be due to the accumulation of ApoB, proliferating cell nuclear antigens (PCNA) in smooth muscle cells and increased endothelial cell permeability [25].

Diagnosis of MB under coronary angiography is based on the typical “milking effect” and a “step down-step up” phenomenon induced by muscle compression during systole [6]. Though coronary angiography is now the gold standard and is most widely used in diagnosing MB, it has some technical restrictions compared with other new imaging techniques, such as intravenous ultrasound (IVUS), intracoronary Doppler ultrasound, multi-detector computed tomography, and intracoronary pressure devices [1, 3, 7]. In other words, the percentage of MB varies with different diagnostic method and equipment. In this retrospective study, the overall incidence of MB was 8.53%, but the female had higher morbidity of MB than the male (10.75% vs. 7.31%, *P* <  0.0001). In the non-SOCAD group, the rate of MB was much higher than that in the SOCAD group (16.05% vs. 1.84%, *P* <  0.0001); whereas, in patients with MB, the rate of SOCAD requiring treatment with PCI or CABG was much lower than that in patients without MB (11.42% vs 56.76%, *P* <  0.0001). Take this in account, we speculated that MB might produce a potential positive role against severe obstructive atherosclerosis in the whole coronary artery system. Accordingly, we analyzed the relationship between MB and severe obstructive atherosclerosis by adjusting for age, sex, hypertension, impaired glucose metabolism, hyperlipidemia, ischemic cerebrovascular diseases, and chronic kidney diseases. Based on our results, there seemed to be a clue that MB might produce a potential protective element against severe obstructive atherosclerosis in the whole coronary artery system (log-OR = − 2.134; *P* <  0.0001).

Hyperlipidemia is a significant risk factor of CAD, which is a wide-accepted truth [28]. In the present study, however, we observed that the rate of hyperlipidemia in SOCAD group was lower than that in non-SOCAD group. We must mention that, it is not interpreted from our result that hyperlipidemia is negatively associated with severe obstructive CAD because of the reasons that patients without SOCAD didn’t receive intensive lipid-lowering management, whereas patients with SOCAD (some of them had a long history of coronary heart disease) received an intensive lipid-lowering therapy even before admission according to the current guidelines.

Although the possible mechanisms of atherogenic protection of MB is unknown, there is still some supported evidence. Loukas et al. [18] found that the bridged segments demonstrated weaker proliferative activities of Ki-67 (a cellular marker for proliferation), and a decreased count of smooth muscle cells and macrophages. This phenomenon might be explained with that the MB-related contracting myocardium compression stimulates the release of anticoagulant and growth factors, which could produce a synergistic effect in preventing the endothelium from denudation, inflammation, and resultant atherosclerosis in vessels with MB and possibly in the whole coronary system. In addition, multi-slice CT scanning showed that the presence of MB was associated with a lower Agatston Calcium Score in the bridged segments [29]. The presence of an MB may also influence arterial tissue through the alteration of hemodynamic forces. According to previous study [24], any atherosclerosis in the MB-segment is suppressed histopathologically and ultrastructurally. Abrupt changes of endothelial cell morphology in the intima beneath the bridge were observed with scanning electron microscopy, which indicates that the arterial tissue beneath the bridge is protected by hemodynamic factors. In cholesterol-fed rabbits, the intima in the MB segment covered by myocardial tissue was free of atherosclerotic lesions, and the endothelial cells were spindle-shaped and engorged [25], which also indicates that the protective element of MB against atherosclerosis might be linked with an alteration of endothelial permeability due to hemodynamic force changes tending towards a higher shear stress. Based on the documented studies as above, the role of myocardial bridges to suppress coronary atherosclerosis might be potential, but it still deserves further scientific research in biochemical and pathophysiological fronts.

Despite the presence of MB can be associated with various complications such as angina, acute myocardial infarction, arrhythmias, and even sudden death [4, 9–17], it can also be considered a benign variation of coronary arteries [19]. So, the treatment of MB is still uncertain due to the lack of convincing evidence. In clinical practice, beta-blockers are usually the first choice of treatment in symptomatic patients [30], other treatments including coronary stents and surgical interventions such as myotomy or bypass are also considered a second-line option. According to a recent systematic review and pooled analysis raised by Enrico Cerrato and colleagues [31], patients with symptomatic isolated MB generally have a good long-term prognosis; pharmacological treatment alone, especially with beta-blockers, can improve angina in most cases. In other words, their study clearly supports that MB is a benign variation of coronary arteries.

### Limitations of this study

There are some limitations in our study, including its non-randomization because of retrospective nature and lack of standardization when MB was diagnosed with coronary angiography. Considering the unreliability of patient’s subjective statement, smoking and family history for CAD, two major risk factors for CAD, were not included in the present study. Furthermore, it is also difficult for us to interpret the exact mechanisms of the potential of MB against severe obstructive atherosclerosis in the whole coronary artery system.

## Conclusions

In conclusion, our results provided a clue that MB might be acted as a potential protective element against severe obstructive atherosclerosis in the whole coronary artery system by adjusting for sex, age, diabetes mellitus, hypertension, and other risk factors, but it still needs further scientific research due to lack of convincing evidence.

## Abbreviations

CABG: Coronary artery bypass grafting
CAD: Coronary artery disease
CKD: Chronic kidney disease
DM: Diabetes mellitus
ICVD: Ischemic cerebrovascular disease
IGT: Impaired glucose tolerance
MB: Myocardial bridge
PCI: Percutaneous coronary intervention
SOCAD: Severe obstructive coronary artery disease
